# Evidence for subjective values guiding posture and movement coordination in a free-endpoint whole-body reaching task

**DOI:** 10.1038/srep23868

**Published:** 2016-04-07

**Authors:** P. M. Hilt, B. Berret, C. Papaxanthis, P. J. Stapley, T. Pozzo

**Affiliations:** 1 INSERM-U1093, Action Cognition et Plasticité Sensorimotrice, Univ Bourgogne-Franche-Comté, Dijon, France; 2 CIAMS, Univ. Paris-Sud, Université Paris-Saclay, 91405 Orsay Cedex, France; 3 CIAMS, Université d’Orléans, 45067, Orléans, France; 4 Neural Control of Movement Lab, School of Medicine, Faculty of Science, Medicine and Health, University of Wollongong, Wollongong, NSW, Australia; 5 Department of Robotics, Brain and Cognitive Sciences, Istituto Italiano di Tecnologia, Genova, Italy; 6 Italian Institute of Technology CTNSC@UniFe (Center of Translational Neurophysiology for Speech and Communication) Via Fossato di Mortara, 17/19 - 44100 - Ferrara; 7 Institut Universitaire de France, Université de Bourgogne, Campus Universitaire, UFR STAPS Dijon, France.

## Abstract

When moving, humans must overcome intrinsic (body centered) and extrinsic (target-related) redundancy, requiring decisions when selecting one motor solution among several potential ones. During classical reaching studies the position of a salient target determines where the participant should reach, constraining the associated motor decisions. We aimed at investigating implicit variables guiding action selection when faced with the complexity of human-environment interaction. Subjects had to perform whole body reaching movements towards a uniform surface. We observed little variation in the self-chosen motor strategy across repeated trials while movements were variable across subjects being on a continuum from a pure ‘knee flexion’ associated with a downward center of mass (CoM) displacement to an ‘ankle dorsi-flexion’ associated with an upward CoM displacement. Two optimality criteria replicated these two strategies: a mix between mechanical energy expenditure and joint smoothness and a minimization of the amount of torques. Our results illustrate the presence of idiosyncratic values guiding posture and movement coordination that can be combined in a flexible manner as a function of context and subject. A first value accounts for the reach efficiency of the movement at the price of selecting possibly unstable postures. The other predicts stable dynamic equilibrium but requires larger energy expenditure and jerk.

When reaching an object from a standing position the brain has to deal with two subtasks^1^: to choose a suitable hand trajectory toward the target and to conserve equilibrium. Reaching out to touch something that is slightly beyond arm’s length and prioritizing the conservation of equilibrium may have an impact on the execution of the arm movement. Alternatively, prioritizing the formation of an accurate hand trajectory over balance control may lead to postural instability. As in this example, human daily interaction with the environment, requires a number of decisions about the strategies to adopt, where some values (or rewards) favor the choice of one solution among many possible theoretic ones. Classically, the decision-making process has been investigated using objective rewards where subjects were paid to perform one task. In these paradigms, subjects compute the utility of each option in evaluating payoffs, costs, or risks^2^,^3^,^4^ to make a rational choice. Humans’ final answers are therefore principally externally driven: the option with the highest payment is the most desirable action. However, in daily life, choosing a hand trajectory to reach an object or how to coordinate posture and movement to push a door may depend mainly upon subjective and homeostatic parameters. The latter type of parameter has rarely been investigated and the implicit values guiding motor responses remain poorly understood^5^. The goal of this study was to understand how subjective values shape the process of deciding upon which motor strategy to adopt. While it is valuable to understand how the processes of deciding upon exogenous, normative and stable offer values (e.g., monetary reward) shape our decisions, such formalism may not be fully adequate to identify implicit sensorimotor values guiding decision making behaviors for voluntary movement. We suggest that previous protocols have only partially addressed the question of free decision-making processes in motor control. Moreover, restricting the decision making process to external criteria (e.g., a salient target) may promote automatic behaviors that map visual stimuli to motor responses^6^. Indeed when pointing towards a salient target the optimal motor solution depends on the spatial localization of that target and less upon free personal choice^7^. The absence of pre-determined reach endpoint, such as when grasping a long stick, instead allows the subjects to freely choose their final hand position^7^,^8^. Consequently, a movement that does not explicitly provide an endpoint to reach towards introduces spatial ambiguity and exposes the subject to a number of subjective and free choices^9^,^10^.

To address these limitations, we designed a protocol where: i) the motor output was not constrained and ii) the reward associated with the different motor choices was not externally but internally driven. If we assume that the behavior adopted reflects these idiosyncratic values, the task could verify whether motor decisions rely upon the exclusion of competing options or on the combination of a few motor preferences. Since the subject was free to choose an upper limb endpoint, equilibrium components may have influenced the motor decision to the greatest extent. Accordingly, current theoretical approaches propose that the coordination of reaching and equilibrium modules follow a hierarchical organization where the perturbation due to upper limb movement is compensated for in advance by a postural component (for a review see^11^). In this case, reach endpoint will be dependent upon a strategy of equilibrium maintenance. Alternatively, if the two components act together to facilitate the execution of the movement^12^ a combination of “competing” mechanisms, trading-off the equilibrium and reaching components of the task, may be predicted.

## Results

**Figure 1:**
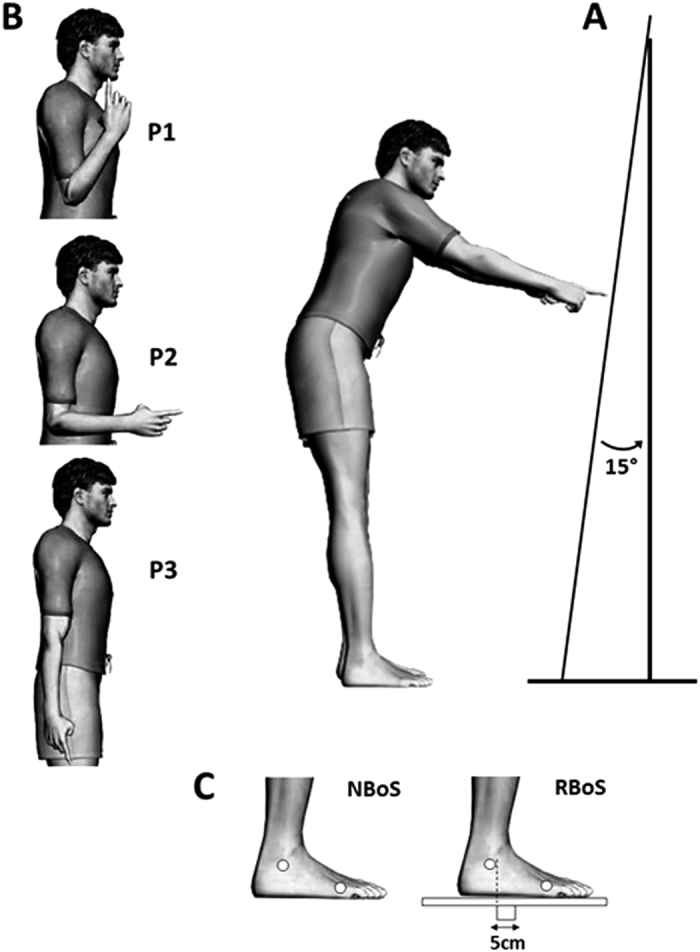
Illustration of the experimental protocol. (**A**) *Experimental paradigm*: subjects had to reach to a homogeneous (without salient point) surface placed at 130% of arm’s length, from the standing position. (**B**) *Representation of the three initial arm starting postures*: P1: hands at shoulder height, elbow bent, P2: forearm at 90° degrees with respect to trunk, and P3: arm hanging vertically along the body (natural posture)). Subjects adopted these initial postures in a random fashion before each trial for each equilibrium condition. (**C**) *Representation of the two equilibrium conditions*: Normal base of support condition (NBoS), subject’s feet normally placed on the floor at a comfortable stance width; Reduced base of support condition (RBoS), in which subjects stood on a horizontal 40 × 40 cm board under which a 5 cm large × 5 cm piece was fixed.

**Figure 2:**
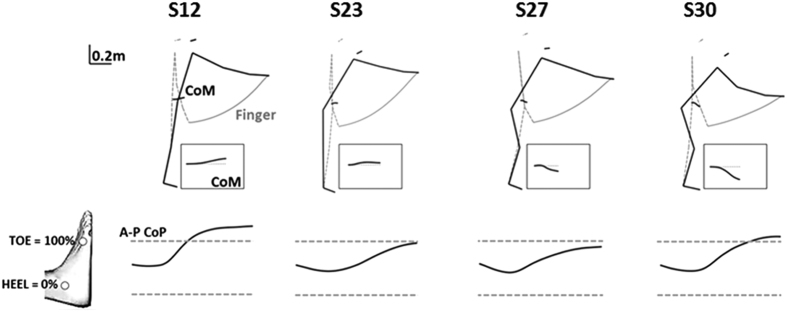
The behaviors of selected subjects. Mean final posture and finger trajectory for four subjects starting from P3. The black box at the lower right of each figure shows the corresponding CoM trajectory (enlarged) relative to the horizontal (dotted gray line). For each typical subject mean CoP expressed as a percentage of toe-heel distance is represented.

The protocol used here (Fig. 1) created considerable inter-individual differences in strategy that led to varying amounts of CoM and finger trajectories as a result of the different joint configurations adopted to achieve the task of reaching to an unspecified target in the NBoS condition. The movements of four exemplar subjects are shown in Fig. 2. Similar finger positions were attained using a variety of body geometry strategies (e.g. S12, S23 and S27). Overall, larger vertical CoM displacements were generally accompanied by a greater amount of joint displacement (e.g., S30). Indeed, a significant correlation emerged between ankle, knee and hip angular displacements and the amount of vertical CoM displacement across all starting postures and for all subjects (respectively *r* = 0.58, *r* = −0.83 and *r* = 0.82, *p* > 0.01). Interestingly, a greater amount of forward (A/P) CoP displacements occurred in the absence of hip/knee flexion (e.g. S12). For subjects shifting the hips backwards with respect to their starting position, A/P CoP displacements ended around the toe marker (e.g. S23, S27 and S30), perhaps to avoid using only the extensor ankle muscle alone to maintain balance at the end of the reach.

Only weak correlations could be found between subject’s movement and anthropometric data (e.g. subject’s height vs final CoM height (in % of subject’s length): R = 0.42 (p < 0.01)). Thus, anatomic variability may partly explain the chosen strategy, suggesting the existence of other parameters influencing subject’s behavior.

**Figure 3:**
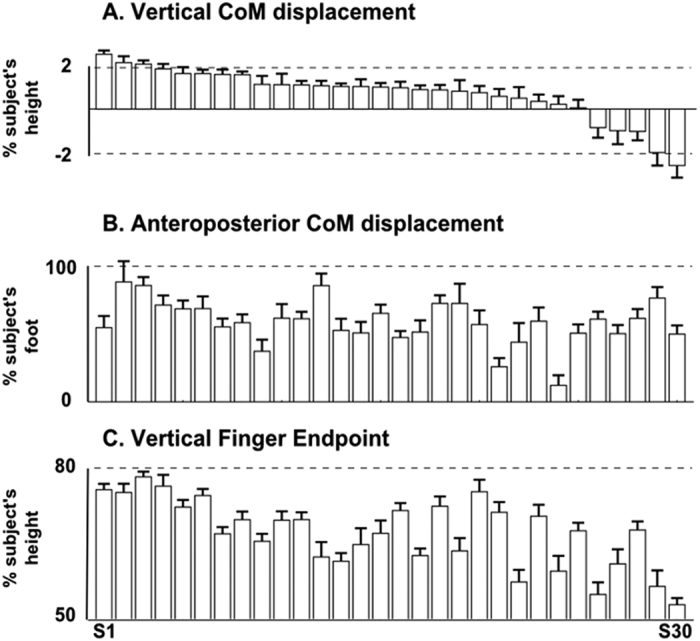
Mean (+/−1SD) centre of mass displacements and finger endpoint positions for each subject. All values are for reaching from posture P3. (**A**) vertical center of mass displacement as a function of subject’s height (0 = CoM starting position), (**B**) antero-posterior center of mass displacement as a function of each subject’s foot length, and (**C**) vertical finger endpoint as a function of each subject’s height. Subjects are ordered in a decreasing manner relative to their vertical center of mass displacement, for the three graphs (**A–C**). Each bar represents one subject.

In an attempt to classify subjects based upon a clear kinematic trend, we used vertical CoM displacement as the reference parameter. This was based upon the finding that vertical CoM displacement was very variable across subjects, ranging from an upward displacement to a downward one. Figure 3 shows all subjects ordered by their vertical CoM displacement (Fig. 3A), positive values being upward displacements, and negative values, downward displacements. It can be seen that, when subjects are ordered by their vertical CoM displacement, each subjects’ vertical finger endpoint follows a decreasing trend (Fig. 3C), whereas A/P CoM displacements do not (Fig. 3B). These observations were confirmed by the significant correlation between vertical CoM displacement and finger endpoint (*r* = 0.74, p < 0.01), while no significant correlation with A/P CoM displacement (*r* = 0.17). Moreover for these three variables, Fig. 3 showed that standard deviation within subject was quite small compare to the inter-subject variability.

The above qualitative observations were quantified for the NBoS condition and are presented in Table 1. Inter-subject standard deviations of mean values of ankle, knee and wrist angles were generally high; superior to the mean values for each and noticeably high for others. Interestingly however, the mean intra-subject variability (across subjects) was relatively small (lower than the inter-subject values) across all final angles, and CoM, CoP and finger displacements. In particular, subjects generally displayed surprisingly low variability in terms of the standard deviation of the endpoint trajectory and the reach endpoint position chosen along the surface. The three initial starting postures led to different mechanical constraints for the movement planning and execution. Table 1 shows little numeric difference between movements starting from the three initial postures. The maximal differences between them, in final vertical finger and CoM vertical displacement and final A/P finger endpoint represent respectively 1.9% (3.3 cm), 0.9% (1.6 cm) and 1.9% (3.3 cm) of average subject height. In order to compare, the length of the reachable region was in average 1.6 m and the standard deviations computed across subjects were respectively: 5.8% (10.15cm), 2.7% (4.72 cm), 1% (1.93 cm). Despite this apparent robustness, we found significant differences between starting postures P1–P3 for a number of measures including: final hip (P1 vs P2), final shoulder (P1 vs P3; P1 vs P2), final elbow (P2 vs P3) and final wrist (P2 vs P1 and P3) angles (see Table 1). To simplify computations, movements starting from P3 will only be considered.

### Impact of a salient target

The presence of a salient target to reach to, located at the average position recorded in the free pointing condition did not significantly influence subject’s behavior in comparison to homogeneous surface reaching. No significant differences (of the 10 subjects tested for this condition) existed between types of target in terms of vertical endpoint finger position (surface: 66.6 ± 7.6%; salient target: 66.7 ± 6.4%, p > 0.05), vertical CoM displacement (surface: 0.68 ± 1.5%, salient target: 0.21 ± 2%, p > 0.05), A/P CoM displacement (surface: 53.1 ± 8%, salient target: 53.9 ± 12.1%, p > 0.05) as well as in the characteristics of the finger movements themselves, for example movement duration (surface: 0.84 ± 0.1 s, salient target: 0.84 ± 0.1 s, p > 0.05), mean hand velocity (surface:1.1 ± 0.1 m.s^−1^; salient target: 1.1 ± 0.2 m.s^−1^,p > 0.05), or the symmetry ratio (surface 0.42 ± 0.1, salient target: 0.40 ± 0.1, p > 0.05). However, finger endpoint variability significantly decreased when subjects reached to a salient target vs only the surface (surface: 1.88%; salient target: 0.42%; F(1, 9) = 41.86, p < 0.01). This difference in finger endpoint could be accounted for by a significant decrease in shoulder angle variability (F(1, 9) = 7.00, p < 0.05). The other angles showed no significant difference between the two equilibrium condition (p > 0.05), suggesting that they are not responsible of the variability observed in finger endpoint.

### Understanding inter individual differences in strategy using optimal control

As detailed in the Methods section, optimal control formalism enables the description of complex whole-body movements in a concise way, by using the optimization of cost functions. Using this approach, we simulated the free-endpoint whole body-reaching task, for each subject and for the starting posture P3, by minimizing five cost functions separately (see Methods). To account for inter-individual differences, we adapted the model by using each subjects’ anthropometric data and movement durations. Distances in joint space between the simulated movements for each cost and the recorded trajectories (see Methods for details) were then quantified, giving a well-suited metric by which to compare recorded and simulated whole movements. Importantly, none of the tested costs could alone account for the whole set of strategies observed, even when modifying imposed constraints on the CoM and CoP.

We found two different cost functions that led to the smallest distances (maximum similarity) across subjects. The first was a combination of the two cost functions that ensured a minimization of energy (absolute work) and smoothness of movement (the integral of the squared angular jerk). We adjusted the coefficients associated with each cost in order to have the two contributing approximately as well as the total sum.


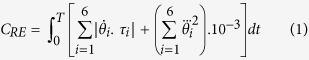


**Figure 4:**
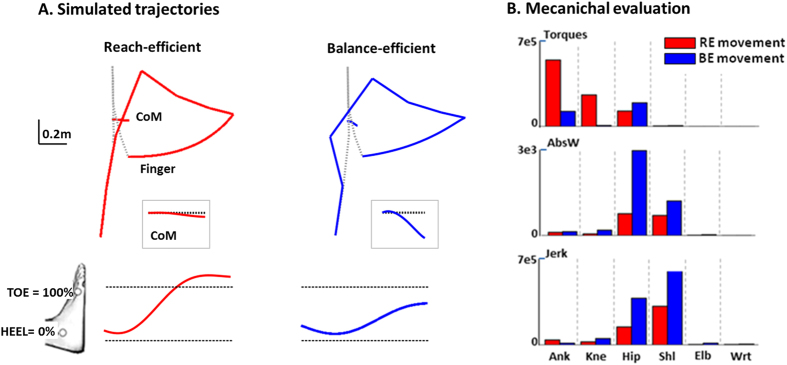
Analysis of movements produced by the two selected costs. (**A**) *Behavior corresponding to the two selected costs*. Mean (across subjects) final and initial angular configurations, and CoM and finger trajectories, obtained for the minimization of each of the two selected costs (balance efficient, blue and reach efficient, red) . The Black box at the lower right shows the corresponding CoM trajectory with respect to the horizontal (dotted gray line). The CoP is represented below each set of stick figures, within foot length (distance from heel to toe markers). (**B**) *Mechanical quantification of simulated movements*. Evaluation of torques (N^2^.m^2^.s), mechanical energy expenditure (N.m) and angular jerk (s^−5^) at each joint (ankle (Ank), knee (Kne), hip (Hip), shoulder (Shl), elbow (Elb) and wrist (Wrt)), for movement simulated using the two selected cost functions (balance efficient, blue and reach efficient, red).

These two costs were already described separately as accounting well for arm reaching movements^13^,^14^,^15^. In particular, this combination of energy/smoothness has previously been shown to characterize the control of free-endpoint reaching in the sitting position^7^. As the cost combinations for our task were similar to those of reaching without equilibrium constraints and because the CoM moved largely forwards with the reach, we now refer to this strategy as being ‘reach-efficient’ (RE, see Fig. 4A, left panel). The second strategy resulted from the integral of the sum of the squared torques and produced downward trajectory of the CoM (Fig. 4A, right panel), increasing equilibrium stability. Moreover, a similar cost was used to model human postural stabilization^16^,^17^. We will refer to it as being ‘balance-efficient’ (BE).

Furthermore, for each cost we analyzed the produced amounts of torque, absolute work and jerk (Fig. 4B, upper, middle and lower rows, respectively) at each joint. These results showed that BE strategy is mainly characterized by a minimization of ankle and knee torques, while neglecting absolute work and jerk principally at shoulder and hip joints, compared to RE. It is important to note that the dichotomy made here between RE and BE costs was inferred a posteriori from simulation results and is dependent from the task used. RE and BE emphasized each one an aspect of the task, but taken individually each cost is able to generate a motor command that handles both equilibrium and reaching at once.

**Figure 5:**
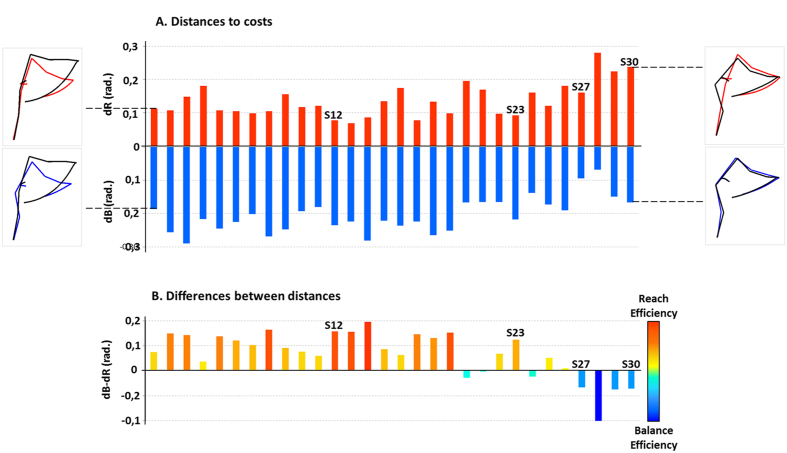
The evaluation of distance from each cost. (**A**) Distances (mean RMSE across angular trajectories) between recorded data and simulated movements using the two selected cost functions (reach efficient: dR, red and balance efficient: dB, blue), for each subject (each bar), in NBoS conditions. Subjects are ordered from left to right based on their vertical CoM displacement (Fig. 2A). The four subjects (S12, S23, S27 and S30) used in Fig. 3 are labeled on the figure. A weak distance to cost means that the behavior of this subject is similar to the movement produced by the minimization of this cost. Mean real movements superposed on simulated movement (adapted to each subject) for the two costs (reach efficient, red and balance efficient, blue) are showed for S1 and S30, respectively at the left and right extremities of the graph. (**B**) Differences between dB and dR for each subject (each bar). The position of the four subjects (S12, S23, S27 and S30) used in Fig. 3 are shown on the figure. Predominance of positive values indicates a preference for reach efficient motor strategies compared to balance efficient ones.

As explained above, to quantify the differences between real and simulated movements (based on RE and BE) for each subject, we computed a metric: mean RMSE values on angular trajectories, named dR and dB respectively (Fig. 5A). Subjects have been ordered relative to their vertical CoM displacement (as in Fig. 3). First, dR tended to show a progressive increase from left to right, suggesting that an upward CoM movement was associated with low dR (i.e., reach efficient behavior) and vice versa. This trend was confirmed by significant negative and positive correlations between dR and dB respectively and the vertical CoM displacement (R = −0.64, p < 0.01 ; R = 0.64, p < 0.01, respectively). Finally, dB and dR both correlated negatively (R = −0.59, p < 0.01) showing that a subject privileging RE (low dR) will tend to neglect BE (large dB) and vice versa.

To better illustrate the relationship between dR and dB across subjects, Fig. 5B shows the difference dB-dR for each subject. Among the 30 subjects tested, 23 (76%) showed positive values indicating a behavior closer to RE than BE (dR > dB), while 7 subjects (23%) showed negative ones (BE behavior: dB > dR). However, we observed varying dB-dR levels, that confirmed the presence of several motor strategies, suggesting that subjects’ behavior cannot be described as two distinct groups, but rather as different distances from the two costs characterizing two extreme behaviors.

### The Impact of reducing the base of support on motor strategies

**Figure 6:**
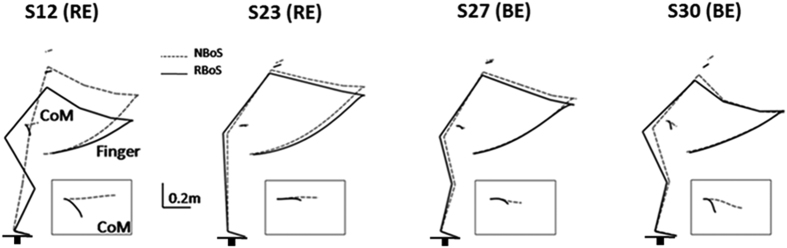
Effect of increasing equilibrium constraints on subject’s behavior. Mean final posture and finger trajectory for the four subjects presented in Fig. 3, starting from P3, in NBoS (dotted line) and RBoS (black line) conditions. Black boxesatthe lower right of each the stick figures represent the corresponding CoM trajectories (enlarged). The reduced base of support is schematically represented under the feet of each subject.

During experiments, we confirmed that subjects could reach the same height in the RBoS condition than in NBoS at the cost of reducing their mean movement speed (NBoS: 1.14 ± 0.2, RBoS control: 0.73 ± 0.2, RBoS: 0.76 ± 0.2, from P3). This control ensured that changes observed in RBoS are not the basic consequence of biomechanical constraints but rather a subject’s specific choice. Figure 6 depicts the average behavior, in RBoS (black lines) and NBoS (dashed lines), for the four representative subjects illustrated in Fig. 2. In order to complete the task in RBoS, all subjects used a degree of backward hip displacement, which limited the A/P displacement of the CoM. Those subjects that moved their CoM downwards in the NBoS condition (i.e., BE behavior, e.g. S27 and S30) kept approximately the same joint configurations in RBoS. Subjects such as S23, who moved their CoM upwards in the NBoS condition (i.e., RE behavior) but did use a degree of hip flexion to limit its forward displacement, preserved their previous joint configurations to achieve the task in RBoS. However, subjects such as S12, who previously moved their CoM upwards (RE behavior) and displaced their CoM to the A/P limit of their BoS, greatly modified their motor strategy to adapt their posture to the RBoS condition.

The principal kinematic parameters, for subjects’ movements recorded in RBoS, are reported in Table 2. CoM vertical displacement increased (F(1, 29) = 29.83, p < 0.01), whereas A/P displacement decreased (F(1, 29) = 155.96, p < 0.01), compared to the NBoS condition (Table 1) and vertical reach endpoint location also decreased significantly (F(1, 29) = 44.96, p < 0.01). Angular values differed, with significant greater knee (F(1, 29) = 15.94, p < 0.01) and hip (F(1, 29) = 50.13, p < 0.01) flexion, and smaller shoulder elevation (F(1, 29) = 17.54, p < 0.01) in the RBoS condition. Moreover, inter-trial variability remained as consistent as in NBoS. Movement velocity (F(1, 29) = 13.01, p < 0.01) and duration (F(1, 29) = 34.09, p < 0.01) decreased and increased respectively, as a consequence of the greater equilibrium constraints. Also time to peak velocity (symmetry ratio) increased significantly in RBoS (F(1, 29) = 13.01, p < 0.01), revealing a longer deceleration phase.

**Figure 7:**
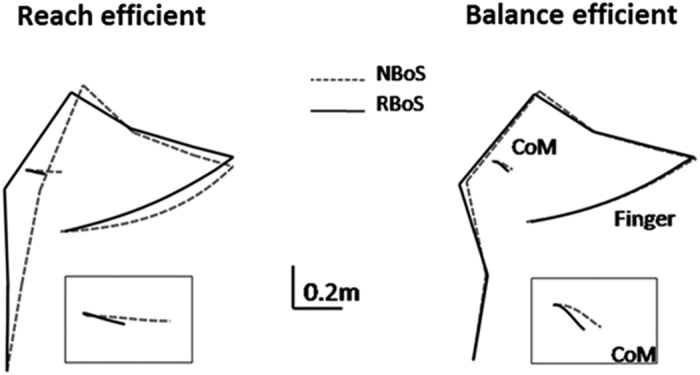
Effect of reduced base of support on simulated movements. Mean final angular configurations and CoM and finger trajectories produced by the minimization of the two selected cost functions, reach efficient (left) and balance efficient (right), in RBoS (black lines) and NBoS (dotted gray lines) conditions. Boxes at the lower right show the corresponding CoM trajectories (enlarged).

In order to evaluate the plausibility of the two selected costs in the face of new equilibrium constraints, we computed new simulations for these costs incorporating the reduced base of support constraint. Figure 7 depicts the reaching movement generated by the optimization of the two costs in the RBoS (black line) and NBoS (dotted line) conditions. As observed with the recorded data (Fig. 6), the two simulations produced a backward hip displacement and a significant CoM downward motion. In BE simulated movement, we observed a preservation of angular configurations adding only a slight backward hip movement (as observed in S27 and S30). Differently, in RE simulated movement a drastic change was necessary producing larger hip backward movements to limit CoM A/P displacement while keeping the CoM relatively high along the vertical axis (as observed in S18).

**Figure 8:**
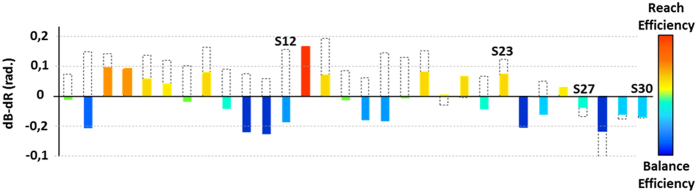
Effect of equilibrium demand on individual motor responses. Differences between distances computed for the two selected costs (balance efficient, d_R_B and reach efficient d_R_R), for each subject (each bar), in the RBoS condition. Values obtained for each subject in NBoS (see Fig. 6) are shownas dotted bars. The positions of the four subjects (S1, S7, S20 and S25) used in Fig. 3 are shown. Negative values indicate a preference for balance efficient motor strategies compared to reach efficient ones.

To quantify the changes observed in individual motor responses due to equilibrium demands, we relied upon the same metric but compared the recorded trajectories in RBoS with the ones predicted by the two costs (RE (d_R_R) and BE (d_R_B)) in the same equilibrium condition. Figure 8 shows the difference d_R_R-d_R_B for each subject. Dotted bars represent the difference computed in NBoS (values showed in Fig. 5B). It can be seen that, while subjects adopted mainly a RE solution in NBoS (23 subjects, 76%), they performed the task in RBoS using mainly a BE solution (19 subjects, 63%). This effect was significant considering the distances d_R_R and d_R_B: on average across subjects, the whole-body motor strategies were significantly less reach-efficient (dR: 0.62 ± 0.04 rad, d_R_R: 0.85 ± 0.08 rad [F(1, 29) = 6.15, p < 0.05]) and more balance-efficient (dB: 0.75 ± 0.04 rad, d_R_B: 0.63 ± 0.05 [F(1, 29) = 5.39, p < 0.05]) in RBoS compared to NBoS.

As observed in Figs 5 and 8, different adjustments were possible to maintain balance under the new equilibrium constraints. In term of distances from the two costs, one may have predicted that subjects adopting a near full RE solution would have kept a RE solution even in the RBoS condition. In contrast, subjects with a mixed solution may have adopted a more BE behavior to maintain balance. However, while BE subjects continued to use a BE solution, RE subjects in NBoS could conserve the RE state in RBoS (7 subjects, 30%), or adopt a more BE behavior (16 subjects, 70%), independently of their distance to the RE cost computed in the NBoS (dotted bars, Fig. 8). To confirm these observations, we found no significant correlation between dB, dR and d_R_B d_R_R, respectively.

## Discussion

Our results showed that a free-endpoint reaching task led the subjects to adopt quite different final whole-body postures, suggesting the presence of idiosyncratic values influencing motor planning. Two principal and complementary optimality criteria (possibly reflecting internal values) explained these differences. Here we discuss the necessity of considering extrinsic redundancy in the motor decision paradigms, and the nature of the internal values extracted.

Subjects produced quite different but consistent movements when faced with multiple choices, due to the large extrinsic (the lack of visible salient target to reach to) and intrinsic (the whole body multi-joint system) redundancy. In contrast, when reaching to a salient target, behaviors have been shown to be more consistent between subjects^18^,^19^. Using optimal control, we found two costs, which replicated the two extreme strategies along a continuum of solutions adopted by the 30 participants. One cost function was based on the combination of mechanical energy expenditure minimization and joint smoothness maximization, while the other one minimized integrated squared muscle torques.

Movements reconstructed using the first function involved mainly the ankle and shoulder joints while freezing knee and hip joints, a strategy which induced a significant horizontal forward CoM displacement and a relatively high reach endpoint. In contrast, the second strategy involved substantial knee flexion and forward trunk bending associated with a backward hip displacement that limited CoP and CoM displacements and led to a relatively low reach endpoint (Fig. 4). As the first function has already been shown to replicate hand reaching from a sitting position (Berret *et al*.^7^) we named it “reach efficient”. The second function inducing limited forward CoP/CoM displacement was labeled “balance efficient”. Furthermore, when equilibrium constraints increased (RBoS), most of the participants who favored a RE solution in NBoS switched to a BE solution, thereby confirming the role of this cost in equilibrium maintenance.

This distinction between RE and BE strategies is reminiscent of the well-known “ankle” versus “hip” strategies observed in postural control tasks^20^,^21^. These postural control strategies were linked to joint torques^22^ although often assuming a hierarchy between reaching and equilibrium, where the perturbation due to upper limb movement is compensated for by postural components^11^. Our study extends these results when reaching goals exist and stability is not the only objective of the system. In that case, humans can choose to weight to a lesser degree the stability component and favor energy expenditure or motion smoothness, whereby producing various extents of ankle or hip strategies during whole-body reaching tasks.

When increasing equilibrium constraints, subjects modified the relative importance they assigned to the two subjective goals, adopting predominantly downward CoM motion and BE movement. This reveals the presence of subject-specific, idiosyncratic values guiding posture and movement coordination that can be combined in a flexible manner as a function of context. These results confirm that the free reach endpoint paradigm is an efficient means for revealing individual choices. While part of inter-individual differences could be accounted for by anthropometric differences, the necessity of introducing distinct cost functions to replicate the behavior of individual subjects showed that idiosyncrasy also arose from central differences. Our results therefore suggest that such inter-subject variability may reflect divergent subjective values driving the motor choice under spatial and postural task indeterminacy.

### Motor decisions are driven by internal values

A basic assumption of optimal control theory is that action selection is guided by internal subjective values^23^,^24^. In this view, motor control is a decision-making process^5^ in which action selection depends on the relationship between possible movements and the associated outcomes (rewards or costs). Our study suggests the existence of subjective reach- and balance-related costs, besides objective ones such as target achievement, which account for the decisional process underlying free reaching while standing.

This trade-off could be performed via the cortex-basal ganglia network that shapes the decision process to satisfy desirable physiological values^25^. Precisely, an internal reward associated with a preferred mix between the two identified cost functions could be reflected by the activation of dopaminergic systems, which are known to be important during motor planning (e.g., bradykinesia in Parkinson’s patients^26^).

Presently, the pertinent variable is not high-order rule-based (e.g., explicit economic consideration) from prefrontal regions^27^, but is related to a selected body state from subcortical areas^28^ showed that the substantia nigra pars reticulata (SNr), a major output nucleus of the basal ganglia, sends projections to the brainstem, midbrain and thalamic structures, and quantitatively determines the direction, velocity, and amplitude of voluntary movements. Additionally, nigral projections to the mesenpontine tegmental region are known to be involved in postural control^29^. The particular role of the descending striatal pathways makes the contribution of the BG a plausible solution in choosing an eventual whole-body configuration.

Thus, risk consideration and likelihood of falling would act as a particular top-down enhancement modulating the behavioral relevance of the motor choice. An example of this top down effect is the stiffening strategy that reduces CoP and CoM displacement in the BE option, also recorded in subjects with increased anxiety^30^,^31^. Such a modulation of motor decision relative to risk was also shown when specifying explicit rewards^32^, with different sensitivity across subjects (from a risk-aversive to a risk-seeking behavior).

Because the basal ganglia have access to higher-order sensory information, and combines proprioceptive, visual and vestibular inputs, the GABAergic outputs from the SNr can represent error signals in higher order postural control systems. These top-down signals could specify a whole body CoM trajectory. A contribution of the basal ganglia in driving the decision process during the present task that may involve limited online visual input (due to a lack of a defined endpoint to reach) but emphasizes the use of endogenous (proprioceptive) inputs, is also supported by the finding that striatal activity starts long before self-initiated movements in contrast to a visually triggered task^33^.

At the behavioral level we found that the kinematic parameters and temporal organization were not changed with or without a salient target, suggesting that the planning of the pointing was not visual stimulus dependent. We speculate that final hand position is not decided using a virtual target arbitrarily selected on the surface but via a corresponding forward model of the upcoming whole-body trajectory. This proposal agrees with previous findings^34^ who showed that the reach endpoint gradually emerged from motor planning instead of being sequentially ordered from eye movement toward the visual stimulus. In other terms, extrinsic and intrinsic redundancies would be resolved at once during the planning stage rather than in-series, thereby proving that under such circumstances the reach endpoint emerges from action selection and does not constrain it.

When reaching without target saliency we found that the motor decision was in-between a reach efficient solution and a solution safer for an equilibrium standpoint. These competing internal values are therefore intertwined rather than mutually exclusive, a result that does not corroborate previous “focused selection” models inhibiting unwanted actions and disinhibiting desired actions, with an all-or-none schema^35^,^36^. Competing actions to be excluded should be represented in a sensorimotor map^37^ that links the occipital visual cortex to the motor cortex via the dorsal route^38^. The present strong ambiguity in the visual input may have limited the role of the occipito-parietal dorsal visual stream and explain such a discrepancy. However, a decision process relying on the estimation of the desirability of the RE option relative to BE option, and then the selection of one motor strategy combining the two cannot be excluded.

We additionally found that a reweighting of values was triggered by a change in equilibrium requirements. This is reminiscent of the way the elderly adapt to WBR tasks by exhibiting smaller CoM displacements^39^ consecutively to an energetically expensive ankle muscle co-contraction^40^, compared to young adults. In a stochastic context, as older adults have a decline in sensorimotor function^41^ which inevitably leads to a more uncertain control of equilibrium in a natural BoS configuration, the reduced BoS emulates such a functional deficit for young adults.

## Methods

### Participants

A total of thirty subjects (21 males, age: 24 ± 3 years; mass 70 + 11 kg; height 1.73 + 0.08 m) participated voluntarily in the experiment. All of them were healthy, right-handed, with normal or corrected-to-normal vision and did not receive explicit information about the purposes or hypotheses of the experiment. All subjects were made aware of the protocol, and written consents were obtained before the study. Experimental protocol and procedures were approved by the Dijon Regional Ethics Committee and conducted according to the Declaration of Helsinki.

### The motor task

From a standing position, participants were asked to perform a series of pointing movements towards a homogenous surface upon which no specific reach endpoint was drawn (see Fig. 1). This surface (2.5 high × 1.5 m long) was a uniform opaque curtain fixed to a wooden frame. The surface was soft enough to prevent subjects from using it as a support when breaking the motion, but sufficiently stretched to keep its shape and remain straight at a 15° angle with respect to the vertical throughout the experiment. We chose to place the target surface at a distance shoulder-surface of 130% of arm’s length. Thus, the distance to, and the angle of the surface were adapted to each subject’s arm length. These distance and angle were chosen to allow a significant reaching distance (1.6 meters in average), requiring the controlled maintenance of equilibrium without placing subjects beyond the limits of their balance, possibly requiring a forward step^42^.

The following verbal instruction was given to all participants: “When ready, point to the surface in front of you, using both index fingers simultaneously, at your own chosen speed, using one discrete movement”. Subjects were also told that they could point at any position they chose along the surface. Therefore, the reach endpoint was not specified by the protocol but depended on each subjects’ own decision. Care was taken that subjects performed discrete movements and that they conserved foot position during trials, and none were subsequently discarded through non-compliance of these trial characteristics. Subjects’ were required to move both arms together leading to symmetrical, almost planar (sagittal) movements. Indeed, it has previously been shown that in a similar task the displacements of all markers lay approximately along the para-sagittal plane^18^. For this reason, we chose to study only one side of the body in 2D coordinates during subsequent analyses and modeling.

To reduce the effects of habituation between trials of the same starting configuration and to investigate if initial conditions drove the choice of strategy, we required subjects to begin their movements from three different initial arm postures presented in a pseudorandom order. We defined these initial postures in terms of their angular arm configurations, denoted as P1 to P3 in Fig 1A; P1: Forearms flexed with the hands held at shoulder height, P2: forearm folded at 90 degrees with respect to trunk vertical axis and P3: arm held hanging vertically alongside the body (Fig. 1B). An experimenter verified that for each starting position subjects assumed the same initial posture (arm with respect to the body).

The experiment was composed of two successive experimental blocks corresponding to two different equilibrium conditions (Fig. 1C). In each block, 44 trials per subject were executed (20 pointing movement starting from P3, and 12 starting from P2 and P1). The first block consisted of a normal base of support (NBoS). In this block, subjects had to reach towards the surface without lifting their heels. In the second block of 44 trials, subjects had to reach whilst standing on a reduced base of support (RBoS). The reduced base of support consisted of a 40 cm wide horizontal square fixed on a thin piece of wood (5cm high, 5cm wide, and 40cm in length, Fig. 1C, right). Participants had to balance on the reduced base of support with the vertical projection of the malleolus of their foot aligned with the backward limit of the thin piece of wood while performing the motor task described below. In order to only modify the equilibrium context and to keep the pointing surface distance and angle constant in the RBoS condition with respect to the NBoS one, the pointing surface was raised to the base of support’s height.

We performed two supplemental experiments to confirm the strategies that may be produced under different conditions to salient targets. In the first, 10 subjects reached towards a salient target (one for each arm) attached to the reaching surface at the height of the mean finger endpoint recorded in the first experimental condition (NBoS), starting from the three initial postures. In the second, while standing on the RBoS, 6 subjects reached to an indicated salient target (mean preferred position recorded during the NBoS condition) from the P3 starting posture.

## Data Collection and Processing

### Materials

Whole-body movements in 3 axes (mediolateral, X, antero-posterior, Y and vertical, Z) were recorded using a seven camera motion capture system (Vicon, Oxford, UK) at 100 Hz. Successive positions of 11 retro-reflective markers (15 mm in diameter) were recorded. Markers were placed at the following anatomical locations on the left side of the body: the external cantus of the eye, the auditory meatus, the acromion process, the lateral condyle of the humerus, the styloid process of the ulnar, the apex of the index finger, the D1 vertebral spiny process, the greater trochanter, the knee interstitial joint space, the ankle external malleolus and the fifth metatarsal head of the foot. As mentioned above, for this kind of task movements are largely planar executed in the sagittal plane^18^. We therefore chose to record only the left side of the body. The position of the center of pressure (CoP) was recorded using a force platform (AMTI BP400600, BIOMETRICS France, Gometz-le-Châtel) at a sampling frequency of 1,000 Hz.

### Motion analysis

All analyses were performed using software custom written in Matlab (Mathworks, Natick, MA, USA). Kinematic signals were low-pass filtered using a digital fifth-order Butterworth filter at a cutoff frequency of 10 Hz (Matlab filtfilt function).Movement onset time was defined as the instant at which the linear tangential (Y,Z) velocity of the index fingertip first exceeded 5% of the peak value obtained during the reach movement. The same threshold value was used to detect movement end (when tangential velocity dropped below the 5% threshold). All analyses of reach movement related variables were made during this period. The position of the CoM was calculated using an eight-segment mathematical model consisting of the following rigid segments: head, trunk, thigh, shank, foot, upper arm, forearm, and hand in relation to documented anthropometric parameters^43^. The model used to determine the whole-body CoM position was the same as that previously validated for similar whole-body reaching (WBR) movements^12^. Standard kinematic parameters, already described in arm and WBR studies^18^ were computed and included: movement duration, peak velocity, mean velocity, relative time to peak velocity (defined as the ratio between the acceleration duration and movement duration). Seven intersegmental angles were defined, one for each joint (i.e., ankle, knee, hip, shoulder, elbow, wrist, head). All-time series were normalized to 200 points using Matlab routines of interpolation (e.g., the Matlab spline function).

### Statistical analysis

We used quantile-quantile plots to visually check that the parameters under investigation were normally distributed (qqplot Matlab function). One-way ANOVAs were also performed to analyze the effects of the different conditions on certain movement parameters. When necessary, post-hoc tests were conducted with Tuckey’s test (threshold of significance: 0.01)

## Modeling

### Model of the musculoskeletal system

As explained above, movements lay along the sagittal plane. Therefore, a reasonable approximation was to model the whole-body as articulated rigid bodies with six joints moving in the sagittal plane (at this level, we neglected the head segment as its orientation has little effect on the finger endpoint location). From the classical Lagrangian formalism, it can be shown that the whole-body dynamics of the system can be written as follows:


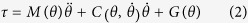


where the variables 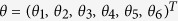 and 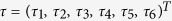 denote the joint angles and resultant muscle torques, respectively. M, C, and G refer respectively to the inertia matrix, the Coriolis/centripetal terms and the Gravitational torques.

These equations are commonly used to describe the mechanics of the musculoskeletal system. We neglected viscous frictions and elastic properties of the tissues as they are difficult to estimate and would introduce a number of uncertain parameters in the simulations. However, even for such a simplified model, computing the dynamics analytically in such away requires long computational time because of the numerous degrees of freedom and is relatively inefficient numerically. To improve efficiency, which is especially crucial to perform optimal control simulations, we used a recursive Newton-Euler algorithm to compute movement dynamics instead. Specifically, we computed system dynamics via the (planar) spatial vector formalism developed by R. Featherstone^44^, whose algorithms are the state-of-the-art for rigid body dynamics. We used the Matlab implementation provided by the author and freely available online.

Rigid-body dynamics were completed using equations that accounted for the low-pass filter properties of skeletal muscles. We considered a simple model of muscle dynamics and assumed the time derivative of muscle torque as the control measure (i.e., a first-order low-pass filter), as per the studies of^45^. The rationale for controlling the rate of change of muscle torque was to capture the smoothness of torque and acceleration profiles as observed during human movement data, especially at the beginning and at the end of the transient motion. Together with the limb dynamics, this formed the control system, denoted hereafter by (Σ).

### Methods of optimal control

The goal of optimal control is to find the movement that minimizes a certain optimality criterion *J* based on task constraints and equations of motion (including subjects’ specific anthropometric data). Mathematically, the goal is to solve the following problem: *to find an admissible control, u and the corresponding admissible trajectory q of the system* (*Σ*)*, connecting a source point, A to a final point on the target manifold B in time T and yielding a minimal value of the cost J*. We considered acceptable a control or a trajectory that satisfied the control or state constraints (respectively) during the entire movement time interval^46^. The state trajectory *q* then refers to the position, speed and acceleration of the six joints angles (see above), 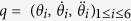 across time. Besides having large dimensions (18-D), the underlying system dynamics is non-linear and putative cost functions may be non-quadratic, which may have made the resolution of such a problem difficult. We used numerical methods (see below) and accurate properties of convergence were obtained through the following procedures; the limb and muscle dynamics together formed a fully-actuated control system (Σ) that could be made linear using feedback. Thus, we could reduce our non-linear dynamics to linear dynamics by actually controlling the derivative of the angular acceleration vector, instead of the derivative of the muscle torque. We then used the angular jerk (i.e. the rate of change or time derivative of the angular acceleration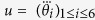) as an abstract control variable. The complexity of the problem was thus left to the cost function, which may be non-quadratic in the control and state variables. For example, it would be quadratic for minimum angle jerk but non-quadratic for minimum torque change (because of Eq. 1). We selected five cost functions, generally used in the motor control literature: ankle torque, sum of torques, torque change, absolute work of torques and angular jerk (Table 3). During our simulations, the source point, A and time, T were matched for each subject with respect to the experimentally recorded mean initial postures and movement durations. Moreover, because subjects had to start and stop their movements in a quasi-static equilibrium state, we considered that, for each simulation, zero angular velocity and acceleration existed at the start and end of the movements. The anthropometric parameters such as moments of inertia and segment masses were derived from documented tables^43^ and the horizontal surface-shoulder distances were set based on their experimental mean values. The latter allowed us to account for experimental variations and physical inter-individual differences during the simulations.

The target manifold, B of the optimal control problem was defined using the following implicit equation:


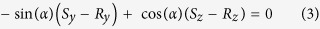


Where *S* = (*S*_*y*_*, S*_*z*_) and *R* = (*R*_*y*_*, R*_*z*_) are the coordinates of the final finger endpoint of the simulated movement and of the mean real finger endpoint of the particular subject, respectively. The parameter, α was the angle of the surface with the vertical (here 15°). To completely formulate the optimal control problem and avoid unrealistic solutions, we also added several constraints to state and control variables. The precision with which the simulated final finger position had to be in the surface equation was 1 cm (terminal constraint). To satisfy biological articular limits, we constrained each joint angle to a realistic range based on maximum and minimum joint angles observed at each joint across all subjects and trials. Velocities, accelerations and jerks were constrained to relatively large values and we verified a posteriori that the boundary values were never attained during simulations. Finally, we added a path constraint to force the CoP and the vertical projection of the CoM to move within the base of support (NBoS or RBoS), thereby ensuring whole-body equilibrium. To locate the CoP in our model, we calculated the muscle torques and forces based on inverse dynamics since we effectively controlled angular jerk during simulations. We computed the A-P CoP position based on the formula described in^47^ from the fundamental principle of static equilibrium applied to the feet (we assumed the feet were fixed as required in the real experiment). Whole body CoM position was deduced from Winter’s table as was the position of the CoM of each moving segment in the sagittal plane^43^. We used the maximal CoP displacement observed in the experimental data, across subjects and conditions, to define lower and upper bounds of the A-P CoP locations. Precisely, this variable had to remain, between −0.05 m and respectively, 1.5 times and 0.8 times the foot length (from malleolus marker to fifth metatarsal) during the entire movement, respectively for NBoS and RBoS (y = 0 being the A-P position of the ankle joint, i.e. the malleolus marker). This was imposed as a nonlinear path constraint in the optimal control problem formulation.

To solve the optimal control problem, we used a direct transcription technique consisting of transforming it into a nonlinear programming problem (NLP) with constraints (optimization problem). To do that, we used the Gaussian pseudo-spectral method to convert the continuous optimal control problem into a discrete problem, and relied upon the Matlab software implementation GPOPS^48^. The resulting NLP problem was solved using the well-established numerical software SNOPT^49^.

### Model versus experimental data comparisons

The main goal of the simulations was to compare real and simulated movements. To do this we computed the root-mean-square error (RMSE) between real and simulated joint angle displacements. This metric was appropriate as it permitted a global description of the movement in terms of space and time.

## Additional Information

**How to cite this article**: Hilt, P. M. *et al*. Evidence for subjective values guiding posture and movement coordination in a free-endpoint whole-body reaching task. *Sci. Rep*. **6**, 23868; doi: 10.1038/srep23868 (2016).

## Figures and Tables

**Table 1:** General movement features in NBoS.

NBoS	Inter-Subject						Intra-Subject		
	Mean			Std			Std		
	P1	P2	P3	P1	P2	P3	P1	P2	P3
Final angle Ankle (deg.)	−4,7	−5,0	**−5,1**	6,3	6,2	**6,5**	1,6	1,6	**1,6**
Final angle Knee (deg.)	4,6	5,1	**5,5**	13,0	13,1	**13,7**	2,7	2,7	**2,7**
Final angle Hip (deg.)	*−23,5*	*−24,9*	**−24,2**	12,7	13,3	**13,8**	3,0	3,1	**3,0**
Final angle Shoulder (deg.)	*95,1*	*93,1*	***93,4***	9,8	10,6	**11,1**	3,1	3,5	**3,1**
Final angle Elbow (deg.)	9,2	*9,8*	***8,6***	7,5	7,5	**7,2**	2,2	2,1	**1,5**
Final angle Wrist (deg.)	*4,5*	*7,5*	***5,9***	7,6	7,8	**7,1**	3,6	3,9	**3,6**
Finger Endpoint (V) (%H)	*69,4*	*67,5*	***67,6***	4,9	5,9	**6,5**	1,6	1,9	**1,9**
Finger Endpoint (AP) (%H)	*55,4*	*55,3*	***54,9***	2,6	2,7	**2,7**	0,6	0,6	**0,6**
CoM Displacement (V) (%H)	*−0,2*	*0,0*	***0,7***	1,0	1,0	**1,1**	0,3	0,3	**0,3**
CoM Displacement (AP) (%F)	60,9	60,7	**58,9**	17,2	15,7	**15,7**	8,2	8,2	**7,7**
CoP Displacement (AP) (%F)	66,1	65,1	**65,6**	23,2	22,7	**23,0**	12,6	13,4	**12,4**
Mean Speed (m.s^−1^)	*0,98*	*0,58*	***1,14***	0,17	0,09	**0,18**	0,09	0,06	**0,10**
Max Speed (m.s^−1^)	*1,97*	*1,16*	***2,21***	0,32	0,20	**0,34**	0,18	0,10	**0,19**
Time to Peak Velocity	0,41	0,41	**0,41**	0,04	0,04	**0,04**	0,04	0,04	**0,04**
Movement duration (ms)	*92*	*98*	***90***	15	15	**14**	8	10	**9**

Means and standard deviations across subjects for the three starting postures (P1, P2 and P3). Finger endpoint (Finger Endpt (V) and (AP)) and vertical CoM displacement (CoM Displct (V)) are expressed as a percentage of subjects height, while anteroposterior displacement of CoP and CoM are expressed as a percentage of subjects foot’ length. Means that were significantly different across initial postures are written in italic.

**Table 2:** General movement features in RBoS.

RBoS	Inter-Subject		Intra-Subj.
	Mean	Std	Std
Final angle Ankle (deg.)	−7, 26	7, 47	1, 97
Final angle Knee (deg.)	20, 52	17, 53	4, 20
Final angle Hip (deg.)	−45, 75	15, 13	3, 38
Final angle Shoulder (deg.)	95, 50	10, 18	2, 88
Final angle Elbow (deg.)	8, 56	6, 98	3, 07
Final angle Wrist (deg.)	6, 33	6, 02	3, 07
Finger Endpoint (V) (%height)	58, 67	6, 07	1, 88
Finger Endpoint (AP) (%height)	52, 95	2, 86	0, 64
CoM Displct (V) (%height)	−1, 73	1, 96	0, 52
CoM Displct (AP) (%foot)	21, 68	7, 41	5, 29
CoP Displct (AP) (%foot)	N.A.	N.A.	N.A.
Mean Speed (m.s^−1^)	0, 83	0, 27	0, 10
Max Speed (m.s^−1^)	1, 69	0, 48	0, 18
Time to Peak Velocity	0, 38	0, 06	0, 05
Movement duration (ms)	1, 17	0, 53	0, 24

Means and standard deviations for all subjects and starting postures.

**Table 3:** Equations of the five tested costs.

Cost	Equations
Angular Jerk	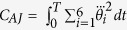
Absolute Work	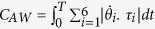
Torque Change	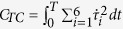
Sum of Torques	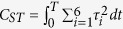
Ankle Torque	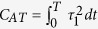
